# Herpes Simplex Virus Hepatitis in an Immunocompetent Host Resembling Hepatic Pyogenic Abscesses

**DOI:** 10.1155/2016/8348172

**Published:** 2016-10-30

**Authors:** Carrie Down, Amit Mehta, Gayle Salama, Erika Hissong, Russell Rosenblatt, Michael Cantor, David Helfgott, Kristen Marks

**Affiliations:** ^1^Department of Medicine, New York-Presbyterian Hospital/Weill Cornell Medical Center, New York, NY 10065, USA; ^2^Department of Radiology, New York-Presbyterian Hospital/Weill Cornell Medical Center, New York, NY 10065, USA; ^3^Department of Pathology, New York-Presbyterian Hospital/Weill Cornell Medical Center, New York, NY 10065, USA; ^4^Division of Gastroenterology & Hepatology, New York-Presbyterian Hospital/Weill Cornell Medical Center, New York, NY 10021, USA; ^5^Division of Infectious Disease, New York-Presbyterian Hospital/Weill Cornell Medical Center, New York, NY 100021, USA

## Abstract

Herpes simplex virus (HSV) hepatitis represents a rare complication of HSV infection, which can progress to acute liver failure and, in some cases, death. We describe an immunocompetent 67-year-old male who presented with one week of fever and abdominal pain. Computed tomography (CT) scan and magnetic resonance imaging (MRI) of the abdomen showed multiple bilobar hepatic lesions, some with rim enhancement, compatible with liver abscesses. Subsequent liver biopsy, however, revealed hepatocellular necrosis, HSV-type intranuclear inclusions, and immunostaining positive for herpes virus type 2 (HSV-2). Though initially treated with broad-spectrum antibiotics, following histologic diagnosis of HSV hepatitis, the patient was transitioned to intravenous acyclovir for four weeks and he achieved full clinical recovery. Given its high mortality and nonspecific presentation, one should consider HSV hepatitis in all patients with acute hepatitis with multifocal hepatic lesions of unknown etiology. Of special note, this is only the second reported case of HSV liver lesions mimicking pyogenic abscesses on CT and MRI.

## 1. Introduction 

Herpes simplex virus (HSV) is a common mucocutaneous infection with specific populations at relatively increased risk of disseminated disease [1], particularly those who are immunocompromised [2] and pregnant women [3]. HSV hepatitis represents a rare complication of HSV infection, which can progress to acute liver failure and, in some cases, even death [4, 5]. HSV hepatitis has been reported to represent less than 1% of all acute liver failure cases and less than 2% of all viral causes of acute liver failure [6]. The present case report concerns a previously healthy, immunocompetent, 67-year-old male who presented with disseminated HSV infection, with both genital and hepatic involvement, and was treated successfully with intravenous acyclovir.

## 2. Case Presentation 

A 67-year-old man with a history of prostate cancer status after prostatectomy 7 years ago, with no residual evidence of disease, and hypothyroidism presented with one week of dysuria and erythema of the ureteral meatus, as well as fever and bilateral upper-quadrant abdominal pain two days prior to presentation. He had presented to his primary care provider at the onset of symptoms and he was treated with a seven-day course of Ciprofloxacin for presumed UTI, although his symptoms did not improve on antibiotics. While he did report fatigue and stress over the past two months, his review of systems was otherwise negative. He denied any sick contacts or recent travel, and there was no history of tobacco, alcohol, or illicit drug use. He is married and he reported monogamous sexual activity with his wife.

On initial presentation, the patient was nontoxic appearing male of normal body habitus with BMI of 23. He was febrile to 38°C but otherwise hemodynamically stable. Physical exam was notable for epigastric tenderness and erythema around the urethral meatus, but there were no vesicles or discharge. Laboratory data at the time of admission was notable for only a mild transaminitis, with AST 100 IU/L (ULN 41 IU/L), ALT 103 IU/L (ULN 63 IU/L), alkaline phosphatase 74 IU/L (ULN 91 IU/L), and total bilirubin 0.9 mg/dL (ULN 1.2 mg/dL) and 0.2 mg/dL direct. The patient did not have leukocytosis, white blood cell count 4,8000 WBC/*μ*L. His urine dipstick showed moderate blood but was otherwise negative. Hepatitis panel was notable for elevated hepatitis A IgG/IgM, with negative hepatitis A IgM, as well as negative hepatitis B and hepatitis C. Blood cultures were negative for bacteria. He was treated with one dose of IV Piperacillin-Tazobactam and Vancomycin upon presentation to the Emergency Room.

**Figure 1:**
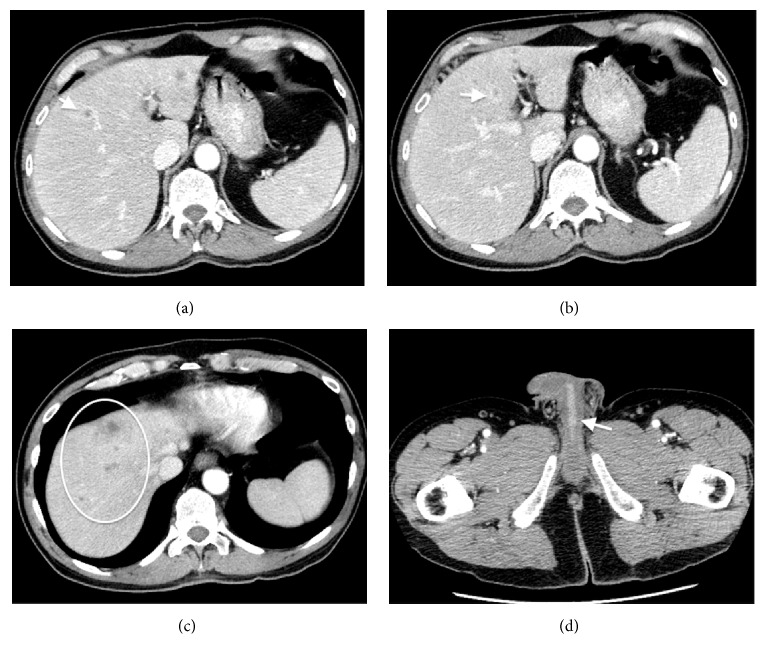
Four axial contrast-enhanced CT images of the abdomen and pelvis. ((a) and (b)) Several rim enhancing lesions are identified in the right and left hepatic lobes (arrows). (c) Nonenhancing hepatic lesions at the hepatic dome (circle); (d) axial image through the inferior pelvis with prominent enhancement along the urethra (arrow).

**Figure 2:**
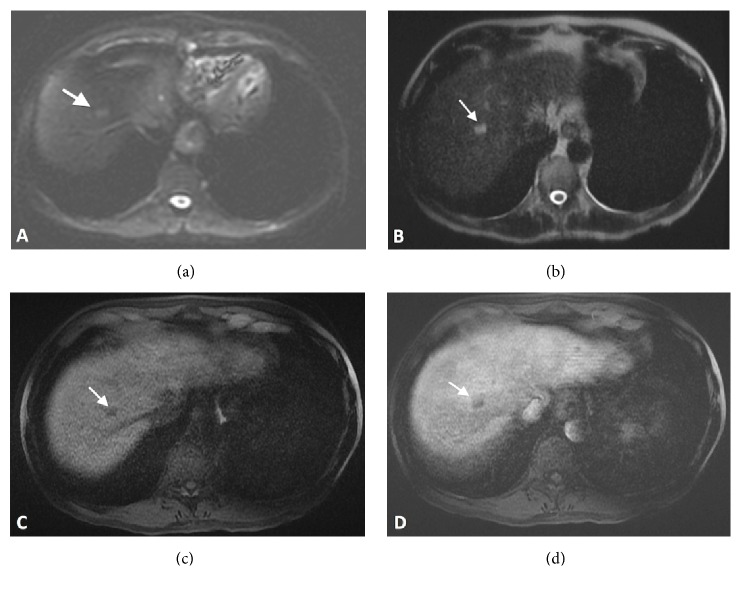
Four axial magnetic resonance images through the liver: (a) diffusion weighted sequence with hyperintense signal in segment 8 lesion showing restricted diffusion, (b) T2-weighted sequence with hyperintense signal within segment 8 lesion, (c) precontrast T1-weighted sequence showing hypointense signal compared to liver parenchyma in segment 8 lesion, and (d) arterial phase postcontrast T1-weighted sequence showing rim enhancement of segment 8 lesion.

Initial CT abdomen/pelvis with contrast demonstrated multiple bilobar hypoattenuating lesions within the liver, some of which have a peripheral enhancement (Figures 1(a)–1(c)). Additionally, there was nonspecific mild edema within the visualized penis with prominent enhancement along the urethra (Figure 1(d)). An MRI abdomen without and with contrast and liver protocol with Eovist was then obtained for further evaluation of the liver lesions. MR images again demonstrated innumerable lesions involving all segments, best delineated on portal venous phase of imaging. The lesions are mildly hyperintense on T2-weighted sequences (Figure 2(a)), demonstrate restriction diffusion (Figure 2(a)), are hypointense on T1-weighted images (Figure 2(c)), and have arterial peripheral enhancement and no washout or central enhancement on delayed phases (Figure 2(d)). Both studies demonstrated lesions that were most compatible with multiple liver abscesses, and histologic sampling and analysis were recommended for further evaluation.

The patient subsequently underwent a CT-guided fine needle aspiration of the left lobe of the liver and became acutely febrile with rigors immediately following the procedure. He was subsequently restarted on broad-spectrum antibiotic therapy with Piperacillin-Tazobactam and Vancomycin for presumed pyogenic hepatic abscesses in the setting of instrumentation.

**Figure 3:**
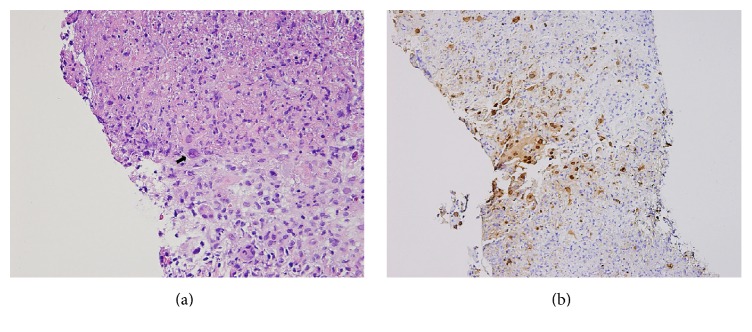
Immunohistochemical analysis of the liver biopsy: (a) high power view showing hepatocellular necrosis. Within the necrotic debris, viral eosinophilic, ground-glass nuclear inclusions can be seen as well as multinucleated cells with nuclear molding (see arrow) and (b) immunostaining for HSV-2 highlights the infected cells.

Gross pathology from the liver biopsy showed hepatocytes with inflammation and necrosis (Figure 3(a)). Pathologic analysis, however, revealed portions of liver parenchyma with necrosis, marked acute and chronic inflammation, and HSV-type cytopathic changes. Within the areas of necrosis, there were numerous cells with intranuclear inclusions. Immunostains for herpes virus type 2 were positive (Figure 3(b)). Studies for acid fast organisms as well as fungal/PCP stains were negative. Stains for herpes virus type 1 and adenovirus were also negative.

Subsequent serologic testing showed elevated HSV-1 and HSV-2 on initial IgG and IgM ELISA testing, with subsequent elevated HSV-2 IgG and normal HSV-1 IgM levels. Quantitative HSV-2 polymerase chain reaction (PCR) of blood demonstrated 200,000 copies/mL. Notably, HIV-1 PCR was negative with normal T-cell subsets. Around this time, the patient also developed painful vesicular lesions on the penis. A swab of the penile lesions was negative for HSV and varicella zoster virus (VZV) on direct fluorescent antibody (DFA) analysis; however, culture was positive for HSV (Table 1).

Given these serologic studies and definitive immunohistochemical results, the patient was diagnosed with HSV hepatitis secondary to HSV-2. Broad-spectrum antibiotics were discontinued and he was transitioned to a four-week course of intravenous acyclovir. His hospital course was complicated by small bowel ileus which progressed to partial small bowel obstruction that is thought to be due to opiate-induced peristalsis reduction that improved with conservative management, and he ultimately achieved full clinical recovery. Repeat CT of the abdomen-pelvis was performed to reassess the small bowel obstruction, and it showed persistence of hepatic nodules.

## 3. Discussion and Conclusion

Disseminated HSV hepatitis is a potentially fatal disorder that accounts for only 0.8% of all acute liver failure cases [6]. The majority of cases involve infection in immunocompromised hosts, including pregnant women in their third trimester and patients with solid organ or hematopoietic transplantation [7]. Our case describes the rare presentation of HSV hepatitis in a previously healthy, immunocompetent adult and highlights the importance of considering HSV hepatitis in all patients with acute hepatitis with multifocal hepatic lesions of unknown etiology. Furthermore, to our knowledge, this is only the second reported case of HSV hepatitis mimicking hepatic abscesses on CT and MRI [8].

As seen in our case, patients can present with a vague, “flu-like” illness most often associated with fever, malaise, and diffuse abdominal pain [4, 9, 10]. Our patient's laboratory results were consistent with the characteristic “anicteric hepatitis” seen in over 90% of HSV hepatitis cases [4, 11, 12]. While significant increases in transaminases (100–1000 fold) are also commonly seen in HSV hepatitis, our patient had only a mild transaminitis.

Interestingly, our case of acute HSV hepatitis presented significantly different imaging findings from those described in previous reports. Characteristic CT imaging features associated with HSV hepatitis include the presence of diffuse hypodense 1 to 3 mm lesions, often confluent, with associated hepatomegaly [13–15]. In contrast to this, our patient's imaging more closely resembled hepatic pyogenic abscesses, typically characterized by larger hypodense lesions or, as seen in our patient, small clustered hypodense lesions with rim enhancement [16]. Following a review of the previous literature, this represents only the second reported case of HSV liver lesions mimicking pyogenic abscesses on CT and MRI [8].

Liver biopsy has historically been the gold standard diagnostic test for HSV hepatitis [7] and it was performed early in the hospital course for our patient. While serologic testing for HSV is limited by a high rate of false-positive and false-negative tests, HSV serum PCR is both a highly sensitive and specific test and can allow for more rapid diagnosis and earlier initiation of empiric therapy if institutionally available. At this time, however, HSV PCR is still largely performed only at reference laboratories with results often not available in time to guide clinical management [7, 17]. In the case of our patient, both liver biopsy and HSV serum PCR were performed. However, while the results of the liver biopsy were available within 3 days of specimen collection, HSV PCR took over 5 days to result. Consequently, if HSV PCR is not institutionally available, our case supports considering liver biopsy early in the clinical course of acute hepatitis.

For the treatment of disseminated HSV, high-dose acyclovir remains the antiviral agent of choice. While there have been no controlled studies evaluating the treatment of HSV hepatitis, intravenous acyclovir 10 mg/kg for up to 21 days is generally recommended [4]. Given its rapid and often fatal course, HSV hepatitis remains an infectious disease emergency that demands early detection and initiation of antiviral therapy. Early treatment with intravenous acyclovir is associated with both increased survival and lower rates of patients requiring liver transplant [2, 3, 18]. In the case of our patient, a prolonged 4-week course of intravenous acyclovir was required prior to clinical resolution.

In conclusion, HSV hepatitis remains a rapid and often lethal disease that, despite being more commonly seen in immunocompromised hosts, can also present in immunocompetent patients. Given the high mortality and nonspecific presentation associated with HSV hepatitis, we recommend the early use of serum HSV PCR, a rapid and specific diagnostic tool. However, if HSV PCR is not institutionally available, we advocate for early liver biopsy in cases of acute hepatitis with multifocal hepatic lesions of unknown etiology. Furthermore, as seen here, the necrotizing liver lesions of HSV hepatitis may be mistaken for pyogenic abscesses on diagnostic imaging modalities.

## Figures and Tables

**Table 1:** Summary of patient's microbiology data.

Test	Result	Normal parameters
Hepatitis Panel		
Hepatitis A IgG/IgM	Ab screen +	Negative
Hepatitis A IgM	Negative	Negative
Hepatitis B core Ab	Negative	Negative
Hepatitis C Ab	Negative	Negative
Serologic Studies		
HSV 1/2 IgM ELISA	467 IV	0.89 IV
HSV 1/2 IgG ELISA	>22.4 IV	0.89 IV
HSV-1 IgG ELISA	0.61	<0.90
HSV-2 IgG ELISA	3.91	<0.09
HSV-2 PCR	200,000 copies/mL	Assay range for HSV 2 is 73 copies/mL to 1 × 10*e*8 copies/mL
Respiratory viral swab	+coronavirus	Negative
HIV-1 PCR	Negative	Negative
CD3+ lymphocytes	846/mm^3^	790–2375/mm^3^
CD4+ lymphocytes	487/mm^3^	387–1688/mm^3^
CD8+ lymphocytes	359/mm^3^	157–856/mm^3^
Penile lesion swab		
HSV DFA	Negative	Negative
HSV culture	Positive	Negative
